# Impact of Cystocele Classification and Surgical Method on Treatment Outcome: A Defect-Oriented Surgical Treatment for Cystocele

**DOI:** 10.3390/jcm15010201

**Published:** 2025-12-26

**Authors:** Pawel Szymanowski, Wioletta Katarzyna Szepieniec, Andrzej Kuszka, Esra Bilir

**Affiliations:** 1 Department of Gynecology and Urogynecology, Faculty of Medicine and Health Sciences, Andrzej Frycz Modrzewski Krakow University, Gustawa Herlinga-Grudzińskiego 1, 30-705 Krakow, Poland; 2 Department of Gynecologic Oncology, Koç University School of Medicine, Istanbul 34450, Türkiye; 3 Department of Gynecology and Obstetrics, University Medical Center Göttingen, 37075 Göttingen, Germany

**Keywords:** cystocele, pelvic organ prolapse, recurrence, sacropexy, apical defect, lateral defect, central defect

## Abstract

**Background/Objectives:** Cystocele remains a prevalent condition with high recurrence rates following conventional native tissue repair. While mesh-based techniques may reduce anatomical recurrence, they are associated with increased complications and regulatory limitations. Our study proposes a defect-oriented approach to cystocele repair to assess whether individualized surgical management based on defect type can improve outcomes, particularly recurrence rates. **Methods:** A single-center retrospective analysis of 317 women undergoing cystocele repair (2019–2020) was performed. Patients were classified into five groups according to defect type: lateral defect at level II, central defect at level II, apical defect, mixed apical and lateral defects at level II, and mixed apical and central defects at level II. Surgical techniques, including vaginal mesh repair, laparoscopic or pre-peritoneal Richardson repair, sacropexy, lateral suspension, and combined procedures, were tailored to the identified defect. Postoperative outcomes and recurrence rates were assessed during follow-up visits. **Results:** The most common defect was apical defect at level II (35.6%) followed by lateral defect (32.8%), mixed apical and lateral (17.7%), central (8.5%), and mixed apical and central (5.4%). The most frequent procedures were vaginal mesh repair (33.8%) and laparoscopic sacropexy (28.7%). In our cohort, the overall recurrence rate was 6.3%, with the highest recurrence observed in the central defect group (11.1%) and lowest in the mixed apical and lateral defect group (0%). **Conclusions:** A defect-oriented classification and individualized surgical approach for cystocele enables effective, durable repair with low recurrence rates. Precise identification of the anatomical defect, rather than the routine use of hysterectomy or mesh, should guide surgical planning to optimize functional and anatomical outcomes.

## 1. Introduction

Pelvic organ prolapse (POP) remains a significant and growing clinical challenge in the field of urogynecology, owing to demographic shifts and increasing life expectancy. Among its various forms, cystocele constitutes the most frequent anterior compartment prolapse; in the Women’s Health Initiative Hormone Replacement Therapy Clinical Trial (n = 27,342), the prevalence of cystocele was found to be 34.3% [1]. The pathogenesis of POP is complex and multifactorial, encompassing anatomical, genetic, obstetric, and lifestyle-related risk factors including age, parity, weight, and obstetric history [2]. POP can be classified using either the Baden–Walker halfway system, the Pelvic Organ Prolapse Quantification (POP-Q) system, or DeLancey’s three-level system [3,4,5]. According to the Baden–Walker halfway system, POP is classified into four stages: grade 0 indicates no prolapse; grade 1, descent halfway to the hymen; grade 2, descent reaching the hymen; grade 3, descent extending halfway beyond the hymen; and grade 4, the maximal degree of prolapse [3]. Compared with the Baden–Walker halfway system, the POP-Q system is more complex, as it assesses pelvic organ support using six defined points (Aa, Ba, C, D, Ap, and Bp) together with measurements of the genital hiatus, perineal body, and total vaginal length, and classifies prolapse into five stages (0–4), from no prolapse to complete prolapse [4].

**Figure 1:**
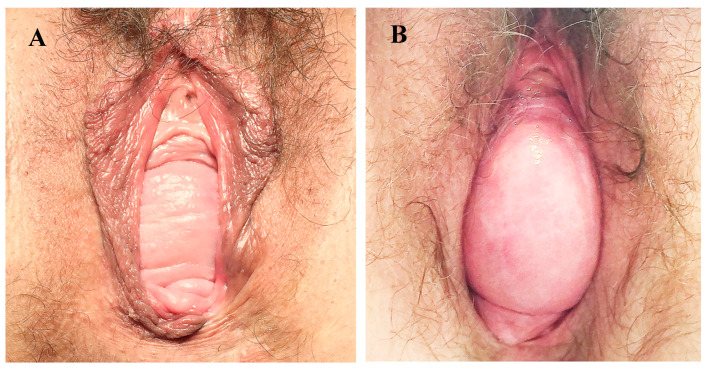
Level II defects in the anterior compartment: (**A**) lateral defect with the vaginal rugae; (**B**) central defect without the vaginal Rugae (Taken from “Uroginekologia. Metody Leczenia Operacyjnego” by Paweł Szymanowski (© Paweł Szymanowski, 2021), published by © Oficyna Wydawnicza Krakowskiej Akademii im Andrzeja Frycza Modrzewskiego, 2021).

DeLancey’s three-level system of pelvic support is widely used to guide surgical planning and categorizes pelvic support defects into three types [5]. Level I defects involve the uterosacral ligaments and may lead to prolapse of the uterus, cervix, or vaginal vault, as well as enterocele formation [5]. Level II defects affect the vesical or rectovaginal fascia and can result in cystocele or rectocele. Level III defects involve the pubourethral ligaments and may cause urethrocele, stress urinary incontinence, or posterior compartment perineal defects [5]. Level II defects in the anterior compartment are additionally divided into central and lateral types: central vesical fascia defects present as cystocele with smooth vaginal mucosa, whereas lateral defects are characterized by preserved vaginal rugae [5] (Figure 1).

Despite widespread use of native tissue anterior colporrhaphy, recurrence remains substantial: in one cohort, 55% of patients (n = 46) in the native tissue group developed recurrent cystocele (≥stage 2), compared to 33% (n = 33) in a mesh-augmented group (*p* = 0.002) [6]. On the other hand, a Cochrane meta-analysis of 37 randomized controlled trials (4023 women) demonstrated that transvaginal mesh repair, while associated with lower anatomical recurrence, was linked to increased rates of reoperation for prolapse or stress urinary incontinence, mesh exposure, bladder injury, and de novo stress urinary incontinence versus native tissue repair [7]. In response to these safety concerns, the U.S. Food and Drug Administration (FDA) mandated in 2019 the cessation of sales of transvaginal mesh for POP repair [8]. However, many European countries still use vaginal mesh in cystocele repair. For instance, a French group conducting a prospective post-2019 study comparing native tissue repair and mesh repair for cystocele, including 52 women in each group, found that native tissue repair did not increase the risk of cystocele recurrence at the 1-year follow-up (rate of stage 2 anterior vaginal prolapse almost 30% in both groups with no statistical difference) [9]. A study from Taiwan including 27 patients undergoing vaginal mesh repair with DynaMesh^®^ reported a 14.8% recurrence rate of stage II cystocele (n = 4) after 3 years of follow-up [10].

The differing outcomes of native tissue versus mesh-reinforced repair highlight an important gap: the focus should shift from the mere choice of material to accurate identification of the underlying defect and individualized surgical strategy. Many surgeons suggest that the high rate of recurrence is attributable less to the choice of mesh per se and more to incomplete understanding of cystocele mechanics, including defects of lateral and central support, as well as the key role of apical support (level I) in anterior compartment prolapse (level II). The functional integrity of the sacrouterine and cardinal ligament complex must be recognized at the surgical planning stage to achieve durable correction [11].

In the present study, we propose a simplified defect-oriented diagnostic and classification algorithm for cystocele, and we evaluate its application in tailoring surgical interventions according to the etiological defect pattern. We hypothesize that such an individualized, mechanism-based surgical approach will lead to improved anatomical and functional outcomes.

## 2. Materials and Methods

This was a single-center retrospective analysis conducted between 2019 and 2020. Institutional Review Board approval was obtained from Andrzej Modrzewski University in Krakow (IRB No.: KBKA50/O/2020). All surgeries were performed by two urogynecologists, each with more than ten years of surgical experience and performing approximately 300 urogynecological procedures annually. All preoperative and postoperative examinations were performed exclusively by one of these urogynecologists.

### 2.1. Preoperative Clinical Assessment

Each patient underwent a standardized urogynecological examination in the lithotomy position, with the urinary bladder moderately filled (approximately 200–250 mL). Kristeller’s double specula were used throughout the examination, and transvaginal ultrasound was employed as part of the qualification process.

The examination began with inspection of the vulva and perineum. Both specula were then inserted to assess the condition of the vaginal mucosa and to visualize either the vaginal portion of the cervix or, in post-hysterectomy patients, the vaginal apex. Patients were instructed to inhale deeply and apply pressure to the pelvic floor. During this maneuver, the specula were gradually withdrawn, and the examiner assessed the degree of descent of anatomical structures at level I (apical support). If no apical defect was identified, evaluation continued at levels II and III of the anterior compartment.

In cases of cystocele, level II defects were categorized as lateral or central (medial). The presence of preserved vaginal rugae indicated a lateral defect, while the disappearance of rugae suggested a central defect (Figure 1) [5]. When an apical defect (level I) was present, its contribution to cystocele formation was evaluated. Using the posterior speculum to reposition the cervix or vaginal apex, the examiner observed whether the cystocele resolved or decreased:

- Complete disappearance of the cystocele indicated that it was caused solely by the apical defect.
- Partial reduction indicated a mixed defect involving level I and level II (lateral or medial).

All findings were documented, including severity based on the Pelvic Organ Prolapse Quantification System (POP-Q) and the presence of urge symptoms or stress urinary incontinence. Pelvic floor sonography supplemented the assessment where it supports the speculum-based examinations such as to diagnose lateral defect. Only patients with cystocele POP-Q stage II or higher were qualified for surgery.

### 2.2. Patient Characteristics

The following baseline characteristics were collected: age, age at first childbirth, gravidity, parity, number of term births, instrumental delivery (vacuum or forceps), cesarean section history, weight of the heaviest newborn, menopausal status, body mass index (BMI), waist-to-hip ratio, primary urogynecological complaint, and relevant obstetric remarks. Medical history included previous surgeries, chronic diseases, hernia, varices, hemorrhoids, asthma (including steroid treatment), smoking status, and family history of urogynecological conditions. Preoperative evaluation documented bladder, urethral, bowel, rectal, vaginal, and pelvic examination findings. If the patients already had symptoms of urge or stress incontinence (SUI grade 1–3), those patients also underwent urodynamic studies. However, it is important to note that cystocele can have a protective effect against incontinence, as it applies pressure on the urethra. Therefore, evaluating for incontinence after cystocele repair is essential, as this may lead to the development of de novo SUI.

In our study, patients with level III anterior compartment defects were excluded. Additionally, patients lost to follow-up or with incomplete medical records or missing essential data were not included in the analysis.

### 2.3. Surgical Planning and Perioperative Care

Cystoceles and corresponding surgical procedures were classified into five anatomical subgroups according to the identified defect. Surgical planning additionally considered patient age, BMI, comorbidities, and patient expectations. Patients with cardiopulmonary contraindications were not qualified for laparoscopic procedures due to pneumoperitoneum and steep Trendelenburg requirements. Postmenopausal patients received estriol 0.5 mg intravaginally every other night for at least two weeks prior to surgery to improve vaginal mucosal quality.

The surgical procedures used in our study included the following:

- Vaginal mesh repair (cranial arms anchored to the sacrospinous ligaments);
- Laparoscopic Richardson lateral repair;
- Pre-peritoneal laparoscopic Richardson repair;
- Anterior colporrhaphy;
- Laparoscopic sacropexy (hysterosacropexy, cervicosacropexy, or colposacropexy);
- Laparoscopic lateral suspension (Dubuisson method) or Noé pectopexy [12];
- Combined procedures: sacropexy with Richardson repair, lateral suspension with Richardson repair, sacropexy with anterior colporrhaphy.

Uterine status guided choice between hysterosacropexy, cervicosacropexy, and colposacropexy.

**Figure 2:**
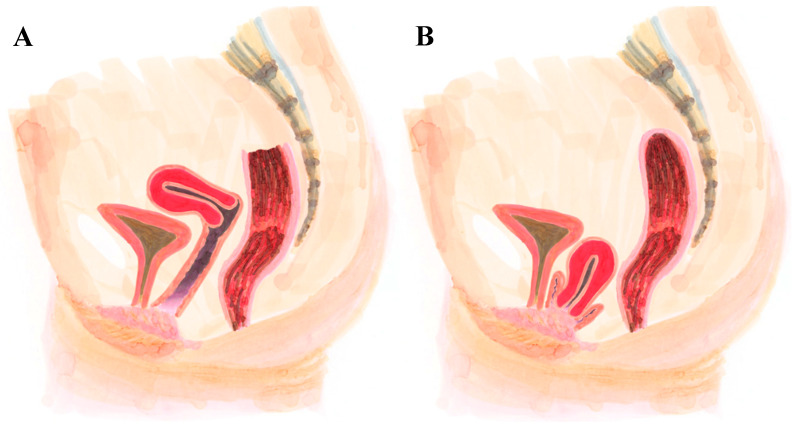
(**A**) Diagram of normal anatomical relationships in the pelvic floor. (**B**) Diagram of apical Defect (Taken from “Uroginekologia. Metody Leczenia Operacyjnego” by Paweł Szymanowski (© Paweł Szymanowski, 2021) published by © Oficyna Wydawnicza Krakowskiej Akademii im. Andrzeja Frycza Modrzewskiego, 2021).

We summarized our proposed defect-oriented surgical strategy as seen in Table 1. Severity after apical repositioning determined whether level II correction was required. In Group 3 cystocele, we included vaginal mesh repair only when laparoscopic surgery was contraindicated; however, this approach remains suboptimal because it causes a posterior deviation of the vaginal axis (Figure 2).

### 2.4. Follow-Up and Outcomes

Postoperative evaluation occurred at 2 weeks, 6 weeks, and 6 months using speculum examination and pelvic floor ultrasound, followed by annual assessments. Complications documented included recurrence (i.e., relapse), hematoma, mesh erosion, defecation disorders, pain, stress urinary incontinence, urinary retention, and other adverse events. Urge symptoms were classified as no urge, dry, wet, or mixed at 6-week follow-up.

### 2.5. Statistics

Data entry and statistical analyses were performed using the Statistical Package for the Social Sciences (SPSS), Version 28.0 for Mac OS X (Chicago, IL, USA). Descriptive statistics were reported as frequencies, medians, and percentiles. Normality of distribution was assessed using both the Kolmogorov–Smirnov and Shapiro–Wilk tests; variables were considered normally distributed when the alpha value exceeded 0.05. Normally distributed data were presented as mean ± standard deviation (SD), while non-normally distributed variables were described using median values and the 25th–75th interquartile range (IQR). Graphical data presentations and visualizations were generated using Microsoft^®^ Excel for Mac (Version 16.95.4). Following Schafer’s recommendation, up to 5% missing data was accepted as the maximum threshold for inclusion [13].

## 3. Results

A total of 317 patients were included in this study. The median age was 58 years (IQR 42.5–68), and the median BMI was 25.3 kg/m^2^ (IQR 22.3–27.8). The median age at first parturition was 25 years, and the median parity was 2. The majority (n = 263; 83.0%) had no history of instrumental delivery, and the median birth weight of the largest child was 3650 g. Most participants were postmenopausal (63.4%). Previous urogynecological surgery was infrequent: hysterectomy in 7.9%, transobturator tape (TOT) in 2.2%, and tension-free vaginal tape (TVT) in 0.3%. A history of hernia was noted in 4.1%, varices in 67.2%, and hemorrhoids in 22.1%. Asthma was present in 2.2%, with 1.6% receiving steroid therapy; an additional 2.8% reported steroid use for other conditions. Smoking was reported by 7.6% of patients, and 24% had a family history of urogynecological disorders.

Regarding cystocele classification, 32.8% of patients had a lateral level II defect (Group 1), 8.5% a central level II defect (Group 2), 35.6% an apical defect (Group 3), 17.7% a mixed apical and lateral level II defect (Group 4), and 5.4% a mixed apical and central level II defect (Group 5). Urinary symptoms were frequent: urge symptoms occurred in 55.5% of patients (21.8% wet, 28.4% dry, 5.0% mixed), and stress urinary incontinence in 41.0% (grade 1: 24.0%, grade 2: 13.9%). Full baseline characteristics are summarized in Table 2.

Overall surgical characteristics are shown in Table 3. The most commonly performed procedure was anterior vaginal mesh repair (n = 107; 33.8%), applicable in Groups 1, 2, 4, and 5. This was followed by sacropexy (n = 91; 28.7%) and pre-peritoneal Richardson repair (17.4%). Additional procedures included laparoscopic Richardson repair (2.5%), anterior colporrhaphy (3.2%), laparoscopic lateral suspension using the Dubuisson method (6.0%), and combined laparoscopic sacropexy with Richardson repair (7.3%). Combined laparoscopic sacropexy with anterior colporrhaphy was performed in 1.3% of cases. No patients underwent either laparoscopic lateral suspension with Richardson repair or combined lateral suspension with anterior colporrhaphy. The median operative time was 85 min (IQR 45–125), including concomitant posterior compartment repair when applicable.

Postoperative complications included recurrence in 6.3% (n = 20), hematoma in 2.8%, mesh erosion in 2.5%, defecation disorders in 0.6%, pain in 2.2%, stress urinary incontinence in 3.8%, urinary retention in 1.9%, and de novo rectocele in 4.7%. At six weeks, urge symptoms improved substantially, with 84.2% reporting no urge, while 4.1% experienced dry urge and 4.1% wet urge; no patients exhibited both simultaneously.

Detailed surgical outcomes across the five defect groups are presented in Table 4.

Group 1 (Lateral Level II Defect): Most patients underwent pre-peritoneal Richardson repair (n = 55; 52.9%), followed by vaginal mesh repair (n = 41; 39.4%) and laparoscopic Richardson repair (n = 8; 7.7%). Recurrence occurred in 9.6%. Median age was 47.0 years (IQR 36.3–64.0), BMI 24.2 kg/m^2^, parity 2, and median largest child weight 3600 g. Approximately half were postmenopausal.

Group 2 (Central Level II Defect): Treatment included vaginal mesh in 63.0% (n = 17) and anterior colporrhaphy in 37.0% (n = 10). Recurrence was 11.1%. Median age was 66.0 years (IQR 59.0–72.0), BMI 27.9 kg/m^2^, parity 2, and largest child weight 3700 g. Most were postmenopausal (92.6%).

Group 3 (Apical Defect): Laparoscopic sacropexy predominated (n = 91; 80.5%), followed by laparoscopic lateral suspension (n = 19; 16.8%), and vaginal mesh (n = 3; 2.7%). Recurrence was lowest at 5.3%. Median age was 58.0 years (IQR 49.0–65.5), BMI 24.2 kg/m^2^, parity 2, and largest child weight 3650 g; 68.1% were postmenopausal.

Group 4 (Mixed Apical + Lateral Level II): Treatment consisted of vaginal mesh (58.9%) or sacropexy with Richardson repair (41.1%). No relapses were observed. Median age was 63.5 years (IQR 38.3–71.0), BMI 25.1 kg/m^2^, parity 2, and largest child weight 3715 g; 67.9% were postmenopausal.

Group 5 (Mixed Apical + Central Level II): Most underwent vaginal mesh repair (76.5%), while 23.5% had sacropexy with anterior colporrhaphy. The relapse rate was 5.9%. Median age was 65.0 years (IQR 61.0–70.5), BMI 28.3 kg/m^2^, parity 3, and median largest child weight 3850 g; 94.1% were postmenopausal.

## 4. Discussion

In this study, 317 patients were analyzed, with a median age of 58 years and a median BMI of 25.3 kg/m^2^. The median age at first parturition was 25 years, and the median parity was 2. Most patients (83%) had no history of instrumental delivery, and the median weight of the largest child was 3650 g. The majority were postmenopausal (63.4%), and previous urogynecological surgeries were uncommon. Among the cystocele subtypes, apical defect was the most frequent (35.6%), followed by lateral (32.8%), mixed apical and lateral (17.7%), central (8.5%), and mixed apical and central (5.4%). In our cohort, de novo SUI was 3.8%.

The most commonly performed surgical procedure was anterior vaginal mesh repair (33.8%), followed by sacropexy (28.7%) and pre-peritoneal Richardson repair (17.4%). The median operative time was 85 min. The overall recurrence rate in the cohort was 6.3%. In Group 1 (lateral defect), pre-peritoneal Richardson repair predominated (52.9%) with a recurrence rate of 9.6%. In Group 2 (central defect), vaginal mesh was performed in 63.0% of patients, and this group showed the highest recurrence rate (11.1%) and the highest median BMI (27.9 kg/m^2^). In Group 3 (apical defect), laparoscopic sacropexy was used in 80.5% of cases and had the lowest recurrence rate (5.3%). Group 4 (mixed apical and lateral) was treated with vaginal mesh (58.9%) or sacropexy with Richardson repair (41.1%), and no recurrences were observed. In Group 5 (mixed apical and central), vaginal mesh was used in 76.5%, with a recurrence rate of 5.9%. Postmenopausal patients accounted for the majority in all groups, especially in Groups 2 and 5 (>90%). Overall, laparoscopic sacropexy demonstrated the lowest recurrence, while vaginal mesh was the most frequently applied technique across both isolated and mixed defect types.

Our defect-oriented surgical approach appears to reduce recurrence compared with conventional, non-differentiated anterior repairs. Notably, we observed a reduction in recurrence rates for anterior colporrhaphy compared to literature reports, in some cases by as much as five-fold. Additionally, hysterectomy was not routinely required in the treatment of cystocele. Since the most frequent indication for hysterectomy in POP surgery is apical prolapse, our findings reinforce that uterine preservation is feasible when apical support is restored adequately.

In a previous retrospective study, recurrent cystocele occurred in 55% of patients who underwent anterior colporrhaphy compared with 33% treated with mesh repair (*p* = 0.002) [6]. In contrast, in our cohort recurrence occurred in 5.6% of patients treated with vaginal mesh (n = 107) and 20% in those treated solely with anterior colporrhaphy (n = 10). A study of 302 patients reported mixed apical and lateral defects as the most common type (31.8%), followed by apical defects (30.8%), lateral defects (23.5%), mixed apical and central (9.6%), and central defects (4.3%) [14]. In our cohort, apical defects were most prevalent (35.6%), indicating population or referral pattern differences.

Although our study was not designed to directly compare approaches to anterior colporrhaphy, in Group 5 (mixed apical and central defect), three anterior colporrhaphies were performed laparoscopically and one vaginally. Recurrence occurred only in the vaginally performed case. Prior evidence from minimally invasive paravaginal repair reported a recurrence rate of 2.3% at 12 months [15]. While our sample size was smaller, our results are consistent with the existing literature.

Serati et al. reported an 86% objective cure rate at 5 years of follow-up in a cohort of 104 patients undergoing combined hysteropexy and anterior vaginal native tissue repair for anterior and central compartment prolapse [16]. Although we performed hysterosacropexy rather than hysteropexy in our cohort, our overall recurrence rate in Group 4 and Group 5 was 6.6% and 0.0%, respectively (Table 4), which remains lower than that reported with their combined approach.

In our cohort, three patients in Group 3 (cystocele caused by apical defect) underwent vaginal mesh repair, with no recurrences observed during the study period. However, we emphasize that this is not an optimal treatment for cystocele due to apical defects, as it can cause a posterior deviation of the vaginal axis. A French study including 1359 women who underwent sacrospinous ligament fixation for apical pelvic organ prolapse reported 44 women (3.2%) requiring reoperation for prolapse recurrence [17]. It should be noted that their cohort included posterior compartment repairs as well as both mesh and native tissue procedures, which differs from our more focused study population [17].

Our findings suggest that cystocele management should prioritize precise identification of the underlying support defect and selection of a procedure that specifically addresses that defect, while also considering general health status and patient preferences. Such individualized planning may enhance surgical durability and reduce risks.

Our study includes a large patient population covering all types of cystocele defects. Moreover, limiting the procedures to two surgeons reduces potential inter-surgeon variability. Similarly, the preoperative and postoperative clinical examinations were performed by a single physician, eliminating inter-observer variability as a potential confounding factor. In addition, the extended follow-up period over many months provides valuable long-term outcome data. This study has limitations. Due to its retrospective design, missing data resulted in exclusion of some cases. Variability in subgroup sizes limited the strength of between-group comparisons. Furthermore, this was a single-center study, which may limit generalizability. Our study focuses on anatomical defects rather than functional disorders such as stress urinary incontinence, and therefore we did not report on the surgical management on SUI.

There is a clear need for multicenter, multi-surgeon prospective studies to further evaluate defect-oriented treatment protocols. Our results establish a framework indicating that tailoring the surgical approach to the specific cystocele defect may reduce recurrence and improve long-term outcomes.

## 5. Conclusions

A thorough understanding of the mechanisms underlying cystocele formation and the selection of surgical techniques tailored to the specific anatomical defect enable effective treatment with low recurrence rates. Importantly, hysterectomy should not be regarded as a standard component of cystocele or POP surgery. It ought to be performed only when clear, independent indications exist.

Surgical management should focus on correcting the defect responsible for the prolapse rather than addressing only the clinical presentation. Avoiding hysterectomy in the treatment of cystocele preserves uterine integrity, reduces operative time, limits morbidity associated with unnecessary uterine removal, and may contribute to meaningful cost savings at the healthcare system level.

## Figures and Tables

**Table 1:** Defect-oriented surgical strategy.

Defect Type	Surgical Approach
Lateral Level II	<75 years/primary cases: laparoscopic or pre-peritoneal Richardson repair; ≥75 years or recurrent: vaginal mesh
Central Level II	Primary: anterior colporrhaphy; recurrent/high-risk/≥75 years: vaginal mesh
Apical	Preferred: laparoscopic sacropexy; alternatives (BMI > 30 kg/m^2^ or adhesions): lateral suspension or vaginal mesh
Mixed Apical + Lateral Level II	Sacropexy or lateral suspension + Richardson repair; mesh for recurrent/high-risk/≥75 years
Mixed Apical + Central Level II	Sacropexy or lateral suspension + anterior colporrhaphy; mesh for recurrent/high-risk/≥75 years; level II corrected only if >POP-Q I after apical repositioning

**Table 2:** Baseline characteristics of the patients.

Variable	Number (n = 317)
Age (years) (IQR)	58 (42.5–68)
Age at 1st parturition (years) (IQR)	25 (22–28)
Gravidity (IQR)	2 (1–3)
Parity (IQR)	2 (1–2)
Parturition at term (IQR)	2 (1–2)
Instrumental delivery - No = 0 - Vacuum = 1 - Forceps = 2	- 263 (83.0%) - 7 (2.2%) - 6 (1.9%)
Weight biggest child (gram) (IQR)	3650 (3370–4000)
Postmenopausal - No - Yes	- 116 (36.6%) - 201 (63.4%)
BMI (kg/m^2^) (IQR)	25.3 (22.3–27.8)
Waist-to-hip ratio (IQR)	0.82 (0.75–0.88)
Any previous urogynecological surgery † - Hysterectomy - TOT - TVT - Vaginal urogynecological surgery - Abdominal urogynecological surgery	- 25 (7.9%) - 7 (2.2%) - 1 (0.3%) - 23 (7.3%) - 6 (1.9%)
Hernia history or present - No - Yes	- 304 (95.9%) - 13 (4.1%)
Varices - No - Yes	- 104 (32.8%) - 213 (67.2%)
Hemorrhoids (n = 36 missing) - No - Yes	- 247 (77.9%) - 70 (22.1%
Asthma (n = 36 missing) - No - Yes	- 274 (86.4%) - 7 (2.2%)
Asthma treated with steroids (n = 36 missing) - No - Yes	- 276 (87.1%) - 5 (1.6%)
Steroids taken for other conditions - No - Yes	- 308 (97.2%) - 9 (2.8%)
Smoking - No - Yes	- 293 (92.4%) - 24 (7.6%)
Family history of urogynecological problems - No - Yes - Not announced	- 196 (61.8%) - 76 (24.0%) - 45 (14.2%)
Cystocele lateral defect level II (Group 1 surgery) - POP–Q 1 - POP–Q 2 - POP–Q 3 - POP–Q 4 - Total	- 4 (1.3%) - 90 (28.4%) - 10 (3.2%) - 0 (0.0%) - 104 (32.8%)
Cystocele central (medial) defect level II POP–Q (Group 2 surgery) - POP–Q 1 - POP–Q 2 - POP–Q 3 - POP–Q 4 - Total	- 2 (0.6%) - 17 (5.4%) - 8 (2.5%) - 0 (0.0%) - 27 (8.5%)
Cystocele with apical defect (Group 3 surgery) - POP–Q 1 - POP–Q 2 - POP–Q 3 - POP–Q 4 - Total	- 6 (1.6%) - 81 (25.6%) - 25 (7.9%) - 1 (0.3%) - 113 (35.6)
Cystocele with mixed apical and lateral defect POP–Q (Group 4 surgery) - POP–Q 1 - POP–Q 2 - POP–Q 3 - POP–Q 4 - Total	- 1 (0.3%) - 26 (8.3%) - 26 (8.3%) - 3 (0.9%) - 56 (17.7%)
Cystocele with mixed apical and central (medial) defects (Group 5 surgery) - POP–Q 1 - POP–Q 2 - POP–Q 3 - POP–Q 4 - Total	- 0 (0.0%) - 9 (2.8%) - 7 (2.2%) - 1 (0.4%) - 17 (5.4%)
Cystocele at level II after level I repositioning - POP–Q 1 - POP–Q 2 - POP–Q 3 - POP–Q 4 - Total	- 80 (25.2%) - 23 (7.3%) - 1 (0.3%) - 0 (0.0%) - 104 (32.8%)
Urge * - No - Dry - Wet - Mix (Dry and wet)	- 141 (44.5%) - 90 (28.4%) - 69 (21.8%) - 16 (5.0%)
Stress urinary incontinence * - No - Grade 1 - Grade 2 - Grade 3	- 187 (59.0%) - 76 (24.0%) - 44 (13.9%) - 9 (2.8%)

* Missing n = 1 (0.3%); † multiple surgeries for a patient possible; TOT: transobturator tape; TVT: tension-free vaginal tape.

**Table 3:** Surgical characteristics.

Variable	Number
Surgical options - Anterior vaginal mesh - Richardson surgery * - Pre-peritoneal Richardson surgery - Anterior colporrhaphy - Sacropexy † - Laparoscopic lateral suspension (Dubuisson method) - Laparoscopic sacropexy and Richardson surgery - Laparoscopic sacropexy + anterior colporrhaphy ‡ - Laparoscopic lateral suspension with Richardson lateral repair - Laparoscopic lateral suspension with anterior colporrhaphy	- 107 (33.8%) - 8 (2.5%) - 55 (17.4%) - 10 (3.2%) - 91 (28.7%) - 19 (6.0%) - 23 (7.3%) - 4 (1.3%) - 0 (0.0%) - 0 (0.0%)
Duration (minutes)	85 (45–125)
Complications - Recurrence - Hematoma - Erosion - Defecation disorders - Pain - SUI - Urinary retention - De novo (all are rectocele)	- 20 (6.3%) - 9 (2.8%) - 8 (2.5%) - 2 (0.6%) - 7 (2.2%) - 12 (3.8%) - 6 (1.9%) - 15 (4.7%)
Urge 6 weeks postoperative - No = 0 - Dry = 1 - Wet = 2 - Dry and wet = 3	- 267 (84.2%) - 13 (4.1%) - 13 (4.1%) - 0 (0.0%)

* One case per laparotomy; † one case per laparotomy; ‡ laparoscopic anterior colporrhaphy n = 3; vaginal anterior colporrhaphy n = 1.

**Table 4:** Surgical group analysis.

Group	Surgery (n, %)	Relapse (%)	Age (Years)	BMI (kg/m^2^)	Parity	Weight Biggest Child (Gram)	Postmenopausal (n, %)
1. Cystocele caused by lateral defect at level II	Vaginal mesh (41, 39.4)	5 (12.2)	67.0 (58.0–76.5)	25.5 (23.1–29.2)	2.0 (1.0–2.0)	3675 (3400–4000)	39 (95.1)
	Laparoscopic Richardson (8, 7.7)	1 (12.5)	36.5 (33.3–41.3)	23.5 (19.4–24.2)	2.0 (1.0–2.8)	3450 (3040–3865)	0.0 (0.0)
	Laparoscopic pre-peritoneal Richardson (55, 52.9)	4 (7.3)	39 (35–46)	23 (21.1–26.5)	0.0 (0.0–0.0)	3600 (3400–3942.5)	6 (10.9)
	Total (104)	10 (9.6)	47.0 (36.3–64.0)	24.2 (22.0–27.4)	2.0 (1.0–2.0)	3600 (3400–4000)	45 (43.3)
2. Cystocele caused by central defect at level II	Anterior colporrhaphy (10, 37.0)	2 (20.0)	60.0 (50.5–68.3)	27.8 (25.1–32.6)	2.0 (1.8–2.3)	3615 (3170–3975)	8 (80.0)
	Vaginal mesh (17, 63.0)	1 (5.9)	68.0 (60.0–73.0)	28.1 (26.0–32.9)	2.0 (1.0–2.0)	3700 (3130–4040)	17 (100.0)
	Total (27)	3 (11.1)	66.0 (59.0–72.0)	27.9 (25.9–32.8)	2.0 (1.0–2.0)	3700 (3200–4000)	25 (92.6)
3. Cystocele caused by apical defect	Laparoscopic sacropexy * (91, 80.5)	6 (6.6)	57.0 (46.0–65.0)	24.2 (21.5–26.3)	2.0 (1.0–2.0)	3620 (3200–4027.5)	58 (63.7)
	Laparoscopic lateral suspension (19, 16.8)	0.0 (0.0)	59.0 (56.0–72.0)	24.4 (23.2–30.9)	1.0 (0.0–3.0)	3650 (3500–4000)	16 (84.2)
	Vaginal mesh (3, 2.7)	0 (0.0)	67.0 (55.0–NA)	29.9 (25.0–NA)	2.0 (2.0–2.0)	4250 (4000–NA)	3 (100.0)
	Total (113)	6 (5.3)	58.0 (49.0–65.5)	24.2 (22.0–26.6)	2.0 (1.0–2.0)	3650 (3310–4042)	77 (68.1)
4. Cystocele with mixed apical and lateral defects at level II	Laparoscopic sacropexy with Richardson lateral repair (23, 41.1)	0.0 (0.0)	36.0 (35.0–47.0)	21.7 (20.7–26.0)	2.0 (1.0–2.0)	3750 (3450–4005)	5 (21.7)
	Laparoscopic lateral suspension with Richardson lateral repair (0, 0.0)	0.0 (0.0)	0.0 (0.0)	0.0 (0.0)	0.0 (0.0)	0.0 (0.0)	0.0 (0.0)
	Vaginal mesh (33, 58.9)	0.0 (0.0)	69.0 (64.0–73.5)	26.0 (24.3–27.8)	2.0 (1.5–3.0)	3600 (3500–4000)	33 (100.0)
	Total (56)	0.0 (0.0)	63.5 (38.3–71)	25.1 (22.2–27.6)	2.0 (1.0–2.8)	3715 (3500–4000)	38 (67.9)
5. Cystocele with mixed apical and central defects at level II	Laparoscopic sacropexy with anterior colporrhaphy ‡ (4, 23.5)	1 (25.0)	67.5 (40.3–70.8)	25.4 (24.6–27.7)	2.5 (2.0–3.0)	4000 (3612.5–4057.0)	3 (75.0)
	Laparoscopic lateral suspension with anterior colporrhaphy (0, 0.0)	0.0 (0.0)	0.0 (0.0)	0.0 (0.0)	0.0 (0.0)	0.0 (0.0)	0.0 (0.0)
	Vaginal mesh (13, 76.5)	0.0 (0.0)	65.0 (61.0–70.5)	28.4 (27.2–29.8)	3.0 (2.0–3.5)	3800 (3487.5–4150)	13 (100.0)
	Total (17)	1 (5.9)	65.0 (61.0 (70.5)	28.3 (26.0–29.3)	3.0 (2.0–3.0)	3850 (3525–4057.5)	16 (94.1)

* In one case, there was conversion to laparotomy; ‡ laparoscopic anterior colporrhaphy n = 3; vaginal anterior colporrhaphy n = 1; NA: not available.

## Data Availability

The raw data supporting the conclusions of this article will be made available by the authors on request.
